# Effect of a long-term treatment with a low-dose granulocyte colony-stimulating factor on post-infarction process in the heart

**DOI:** 10.1111/j.1582-4934.2008.00294.x

**Published:** 2008-02-25

**Authors:** Hideshi Okada, Genzou Takemura, Yiwen Li, Takamasa Ohno, Longhu Li, Rumi Maruyama, Masayasu Esaki, Shusaku Miyata, Hiromitsu Kanamori, Atsushi Ogino, Munehiro Nakagawa, Shinya Minatoguchi, Takako Fujiwara, Hisayoshi Fujiwara

**Affiliations:** a Division of Cardiology, Gifu University Graduate School of MedicineGifu, Japan; b Department of Food Science, Kyoto Women's UniversityKyoto, Japan

**Keywords:** GCSF, heart failure, myocardial infarction, remodelling

## Abstract

Although beneficial effects of granulocyte colony-stimulating factor (G-CSF) have been demonstrated on post-myocardia infarction (MI) process, the mechanisms and feasibility are not fully agreed yet. We investigated effects of a long-term treatment with a low-dose G-CSF started 1 day after the onset of MI, on post-infarction process. One day after being made MI by left coronary ligation, mice were given G-CSF (10 μg/kg/day) for 4 weeks. The G-CSF treatment resulted in a significant mitigation of cardiac remodelling and dysfunction. In the G-CSF-treated hearts, the infarcted scar was smaller with less fibrosis and abundant vessels while in the non-infarcted area, hypertrophic cardiomyocytes with attenuated degenerative changes and reduced fibrosis were apparent. These effects were accompanied by activation of signal transducer and activator of transcription 3 (STAT3) and Akt and also by up-regulation of GATA-4, myosin heavy chain and matrix metalloproteinases-2 and -9. Apoptosis of cardiomyocytes appeared insignificant at any stages. Parthenolide, a STAT3 inhibitor, completely abolished the beneficial effects of G-CSF on cardiac function and remodelling with loss of effect on both anti-cardiomyocyte degeneration and anti-fibrosis. In contrast, wortmannin, an Akt inhibitor, did not affect G-CSF-induced benefis despite cancelling vessel increase. In conclusion, treatment with G-CSF at a small dose but for a long duration beneficially affects the post-infarction process possibly through STAT3-mediated anti-cardiomyocyte degeneration and anti-fibrosis, but not through anti-cardiomyocyte apoptosis or Akt-mediated angio-genesis. The findings may also imply a more feasible way of G-CSF administration in the clinical settings.

## Introduction

A number of studies have demonstrated the beneficial effects of granulocyte colony-stimulating factor (G-CSF), a haematopoietic cytokine, on cardiac structure and function following acute myocardial infarction (MI) in experimental models [1–6]. Since the adverse effects of G-CSF are dose-dependent, it is noteworthy that when G-CSF was administered to animals subjected to acute MI in most of those earlier studies, the dosage seems extremely high (*e.g.* 100 μg/kg/day) even when considering the difference in bioavailability between mice and human beings. In addition, the treatment was started at the time of coronary ligation, during reperfusion, or even before infarction. However, there are still numerous patients with acute MI who come too late to the hospital to have the chance for early coronary reperfusion. These would seem to make earlier studies clinically somewhat irrelevant. In the present study, therefore, we examined whether a small dose of G-CSF (10 μg/kg/day) of which administration was begun 1 day after coronary occlusion could beneficially affect post-infarction cardiac function at the chronic stage (4 weeks after MI). G-CSF was instead administered for a long duration (4 weeks) based on a surmise that because myocardial infarct scar is a highly dynamic tissue [7], intervention not only during the acute stage but also during the subacute and chronic stages might significantly affect the post-infarction cardiac remodelling process [8, 9]. We then sought to determine which phenotypic alterations and which signal transduction pathways are critical for the G-CSF-mediated effects on hearts following MI.

Harada *et al.* discovered the mechanistic importance of Janus kinase 2 (JAK2)/signal transducer and activator of transcription 3 (STAT3) pathway activation for beneficial effects of G-CSF on post-infarction cardiac remodelling in mice [3]. The same group, however reported a critical role of activation of phosphatidyl inositol 3-phosphate (PI3K)/Akt pathway for the benefits [5, 6]. Therefore, relative importance is not known between STAT3 and Akt activations.

The present study therefore has two aims: (1) to examine whether treatment with a small-dose G-CSF for a long duration, started 1 day after the onset of MI, beneficially affects the post-infarction process; and (2) to elucidate the mechanisms involved in the G-CSF actions, including phenotype alterations of the post-infarct heart and relative importance between STAT3 and Akt signals.

## Materials and methods

### Animal experiments

This study was approved by our Institutional Animal Research Committee. MI was induced in 10-week-old male C57BL/6J mice (Chubu Kagaku, Nagoya, Japan) by ligating left coronary artery as described previously [9]. We made MI in 40 mice; 30 mice that were still alive 24 hrs after MI. Beginning on the 2nd day after MI (24 hrs after coronary ligation), recombinant human G-CSF (Lenograstim, Chugai Pharmaceutical Co. Ltd., Tokyo, Japan, 10 μg/kg/day) was administered to the mice by subcutaneous injection once a day for consecutive 5 days each week for 4 weeks (*n*= 14). As a control, the same volume of saline was injected in the same manner (*n*= 16). In the sham-operated mice, a suture was passed around the artery but was not tied; they, too, received saline or G-CSF (*n*= 10 each) in the same manner, beginning on the 2^nd^ day after surgery. All mice were examined 4 weeks later.

Next, to assess the extent to which the effects of G-CSF are mediated *via* the JAK/STAT and PI3K/Akt pathways, other mice were administered parthenolide (Sigma-Aldrich, St. Louis, MO, USA, 1 mg/g/day), a STAT3 inhibitor, or wortmannin (Sigma-Aldrich, 16 μg/kg/day), a PI3K inhibitor. Twenty mice surviving on the 2nd day after MI were randomly assigned into the G-CSF plus parthenolide and G-CSF plus wortmannin groups (*n*= 10 each). The inhibitors were administrated in the same manner and at the same time as G-CSF.

### Cell culture

Cardiomyocytes were isolated from 1-day-old neonatal Balb/c mice as previously described, plated at the concentration of 5 × 10^5^ cells/ml, and then incubated in DMEM (Sigma-Aldrich) at 37°C for 48 hrs [10]. The cells were then pretreated with either parthenolide (50 μmol/l for 2 hrs) or wortmannin (10 nmol/l for 0.5 hr), after which G-CSF (100 ng/l) was added and the cells were incubated at 37°C for an additional 24 hrs. In addition, we also knocked down STAT3 and Akt-1 in cardiomyocytes performed with a Validant Stealth RNAi Kit (Invitrogen, Carlsbad, CA, USA); Negative RNAi (Invitrogen) served as a negative control. Briefly, 40 nmol/l of RNAi per 3-cm dish was added to the cultures, and the cells were transfected using Oligofectamin (Invitrogen) according the manufacturer's instructions. After incubating the cells at 37°C for 24 hrs to knockdown expression of the targeted gene, G-CSF (100 ng/l) was added and the incubation was continued at the same temperature for another 24 hrs. In some experiments, a mixture of agonistic anti-Fas antibody (1 μg/ml, Pharmingen, San Diego, CA, USA) and actinomycin D (0.05 μg/ml, Sigma-Aldrich) was then applied for 24 hrs to induce apoptosis [10, 11]. The *in vitro* experiments were done in triplicates and each of them was performed on the different day.

### Physiological studies

Echocardiography and cardiac catheterization were performed before killing as previously described [9].

### Histological analysis

After making the physiological measurements, the mice were killed and the hearts were removed and cut into two transverse slices: the basal specimens were fixed with 10% buffered formalin and embedded in paraffin. Four-μm-thick sections were stained with haematoxylin and eosin, Masson's trichrome and Sirius red F3BA (0.1% solution in saturated aqueous picric acid) (Sigma-Aldrich). Sirius red-stained preparations were used for assessment of fibrosis in infarcted and non-infarcted areas. Quantitative assessments, including cell size, cell population and fibrosis, were performed in 20 randomly chosen high-power fields (HPF, x400) in each section using a LUZEX F multipurpose colour image processor (Nireco, Kyoto, Japan). The area of MI was traced on preparations stained with Masson's trichrome under a light microscope, calculated using the image processor and expressed as μm^2^. Vessels were identified as the lumens outlined by Flk-1-positive endothelial cells on the immunostained sections.

### Immunohistochemistry

Deparaffinized 4-μm-thick sections were incubated with a primary antibody against G-CSF receptor (G-CSFR, Santa Cruz Biotechnology Inc., Santa Cruz, CA, USA), phosphorylated form of Akt (p-Akt, Cell Signaling, Danvers, MA, USA), Flk-1 (Santa Cruz Biotechnology Inc.) or panleucocyte antigen (CD45, Pharmingen), after which they were labelled with diaminobenzidine hydrochloride. In addition, frozen 4-μm-thick sections were incubated with a primary antibody against phosphorylated form of STAT3 (p-STAT3, Cell Signaling). As the nagative controls, sections were incubated with the iso-type immunoglobulins instead of the primary antibodies.

*In situ* nick end-labelling (TUNEL) assay was performed using an ApopTag kit (Intergene Co., Purchase, NY, USA) according to the supplier's instructions. As the negative controls for TUNEL assay, terminal deoxynucleotidyl transferease (TdT) enzyme was omitted.

For double immunofluorescent labelling, tissue sections were stained first using an FITC-conjugated ApopTag kit (Intergene Co.) and then with anti-myosin heavy chain (MHC) antibody (Santa Cruz Biotechnology Inc.) followed by labelling with Alexa Fluor 568. Nuclei were stained with Hoechst 33342.

### Western analysis

Total protein concentrations in tissue or cell lysates were measured using BCA protein assays. Samples of the protein (50 μg) were then separated by 10% SDS-PAGE and transferred onto nitrocellulose transfer membranes (Millipore, Billerica, MA, USA). The membranes were then probed using antibodies against G-CSFR, STAT3 (Chemicon, Temecula, CA, USA), p-STAT3, Akt (Cell Signaling), p-Akt, extracellular signal-regulated kinase (ERK), phosphorylated form of ERK (p-ERK, both from Cell Signaling), atrial natriuretic peptide (ANP), GATA-4 (Santa Cruz, Biotechnology, Inc.), MHC, matrix metallopro-teinase-2 (MMP-2, Daiichi Fine Chemical Co., Toyama, Japan) or MMP-9 (Santa Cruz, Biotechnology, Inc.). The blots were then visualized using chemi-luminescence (ELC, GE Healthcare UK Ltd., Buckinghamshire, UK), and the signals were quantified by densitometry (NIH IMAGE 1.63). Samples of *n*= 3-5 from each group were subjected to Western blotting.

### Electron microscopy

The heart was minced, immersion-fixed overnight with phosphate-buffered 2.5% glutaraldehyde (pH 7.5), postfixed for 1 hr with 1% osmium tetroxide dehydrated through a graded ethanol series and embedded in Epon medium. Ultrathin sections were stained with uranyl acetate and lead citrate and observed in an electron microscope (H700; Hitachi, Tokyo, Japan).

### TUNEL at the electron microscopic level (EM-TUNEL)

EM-TUNEL was carried out as described previously [12], after which the specimens were observed under a Hitachi 700 electron microscope.

### Statistical analysis

Values were shown as mean ± S.E.M. The significance of differences between groups was evaluated using one-way ANOVA with a *post hoc* Newman-Keul's multiple comparisons test. Values of *P* < 0.05 were considered significant.

## Results

### Survival, cardiac function and peripheral granulocyte count at the chronic stage of MI

Although there was no significant difference in the survival rates between the saline (63%, 10 out of 16) and G-CSF (79%, 11 out of 14) groups 4 weeks after MI, echocardiography and cardiac catheterization showed severe left ventricular remodelling with marked enlargement of the left ventricular cavity and signs of diminished function in the control mice. Compared with the saline-treated controls, G-CSF-treated mice showed smaller left ventricular cavities and significantly better left ventricular function (Table 1). G-CSF treatment had no effect on cardiac function in sham-operated mice.

**Table 1:** Effect of G-CSF and inhibitors on cardiac function at 4 weeks after surgery

*n*	Sham + Saline 10	Sham + G-CSF 10	MI + Saline 10	MI + G-CSF 11	MI + G-CSF + Parthenolide 6	MI + G-CSF + Wortmannin 7
LVDd, mm	3.69 ± 0.03	3.70 ± 0.03	5.70 ± 0.01*	5.10 ± 0.01*#	6.37 ± 0.20*^+^	4.78 ± 0.15*#
FS, %	33.8 ± 0.6	35.0 ± 0.7	11.4 ± 1.2*	19.3 ± 1.5*#	13.9 ± 0.6*+	22.9 ± 0.7*#
HR, bpm	538 ± 45	542 ± 20	558 ± 10*	600 ± 16*#	516 ± 12*^+^	535 ± 26*#
LVSP, mmHg	108 ± 2.8	110 ± 2.4	55 ± 2.4*	65 ± 2.7*#	57 ± 2.0*^+^	71 ± 2.1*#
LVEDP, mmHg	0.7 ± 0.2	0.5 ± 0.1	10.0 ± 0.9*	5.2 ± 0.6*#	9.4 ± 1.3*+	4.4 ± 0.7*#
+dP/dt, mmHg/s	8795 ± 459	9126 ± 584	2666 ± 345*	4176 ± 403*#	2894 ± 244*^+^	4881 ± 159*#
−dP/dt, mmHg/s	−8994 ± 466	−9266 ± 509	−2528 ± 264*	−3681 ± 319*#	−2449 ± 215*+	−4435 ± 287*#

MI, myocardial infarction; LVDd, left ventricular end-diastolic diameter; FS, fractional shortening; LVSP, left ventricular peak systolic pressure; LVEDP, left ventricular end-diastolic pressure; *P < 0.05 *versus* saline-treated sham; #*P* < 0.05 *versus* saline-treated hearts with MI; ^+^*P* < 0.05 *versus* G-CSF-treated hearts with MI.

Four weeks later, the granulocyte count in peripheral blood was significantly higher in G-CSF-treated mice (3863 ± 126 cells/μl in sham; 4173 ± 917 cells/μl in post-MI) than in saline-treated mice (932 ± 126 cells/μl in sham, *P* < 0.05; 1123 ± 325 cells/μl in post-MI, *P* < 0.05). No adverse reactions to G-CSF, including splenic rupture, splenomegaly, thromboembolism, intestitial pneumonia and atherosclerosis, were detected.

### Cardiac morphology and protein expression at the chronic stage of MI

**Fig. 1:**
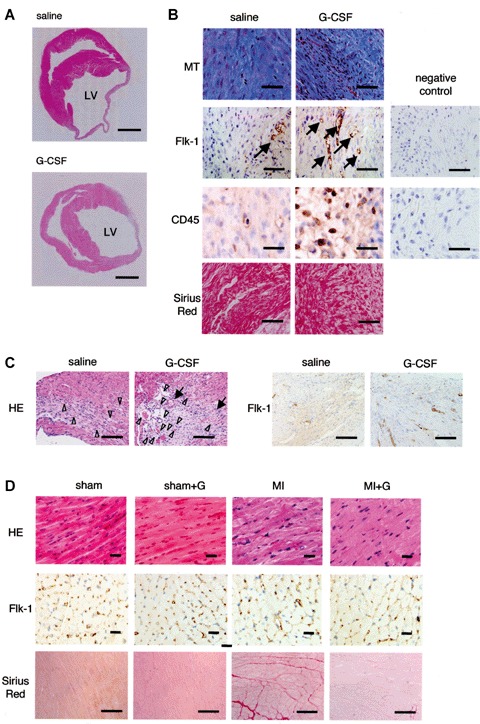

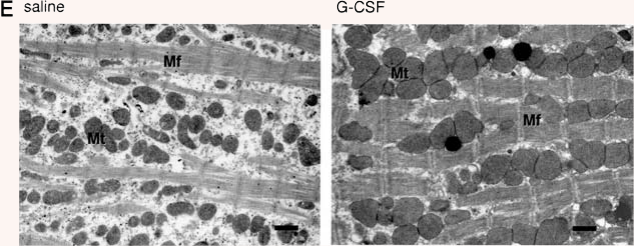
Effect of G-CSF on cardiac morphology 4 weeks after MI. (**A**) Transverse sections of infarcted hearts. Masson's trichrome staining. Bars, 1 mm. (**B**) Histological (Masson's trichrome, Sirius red) and immunohisto-chemical (Flk-1, CD45) staining of the infarcted area of a saline-treated and G-CSF-treated heart. The most right panels show negative control sections for immunostains. Arrows indicate immunopositive cells. Bars, 50 μm in CD45 staining; 100 μm in the others. (**C**) Haematoxylin and eosin and Flk-1 immunohistochemical staining of the border zones between infarcted and non-infarcted area of a saline-treated and G-CSF-treated heart. Arrows, arterioles; arrowheads, venules. Bars, 100 μm. (**D**) Histological staining (haematoxylin and eosin and Sirius red) of the non-infarcted area of hearts. Bars, 100 μm. (**E**) Ultrastructure of salvaged cardiomyocytes. Mf, myofibrils; Mt, mitochondria. Bars, 1 μm.

The MI area was smaller in G-CSF-treated mice (Fig. 1A and Table 2). In control hearts, the infarcted area was replaced with fibrotic scar tissue containing few cellular components, while the non-infarcted area showed interstitial fibrosis. In G-CSF-treated mice, however, populations of Flk-1-positive vessels and leucocytes within the infarcted area were significantly greater (Fig. 1B and Table 2). At the border zone between infarcted and non-infarcted area, the Flk-1-positive vessel population was also significantly greater in the G-CSF-treated group (29 ± 2.6 /HPF) than in the saline group (9.8 ± 1.4 /HPF). So were the number of arterioles (saline, 0.58 ± 0.11 /HPF *versus* G-CSF, 1.5 ± 0.18 /HPF) and that of venules (saline, 1.9 ± 0.30 /HPF *versus* G-CSF, 5.1 ± 0.85 /HPF) assessed on the haematoxylin and eosin-stained preparations (Fig. 1C). In the non-infarcted area, on the other hand, the Flk-1-positive vessel population tended to be greater in the G-CSF-treated group but there was no statistical difference compared with the control (Fig. 1D and Table 2). The ratio of vessels to a cardiomyocyte in the non-infarcted area was similar between the groups (Table 2).

**Table 2:** Effect of G-CSF and inhibitors on cardiac histology at 4 weeks after surgery

*n*	Sham + Saline 6	Sham + G-CSF 6	MI + Saline 6	MI + G-CSF 6	MI + G-CSF + Parthenolide 3	MI +G-CSF + Wortmannin 4
**Infarcted area**						
MI area, x10^5^μm^2^	N.A.	N.A	4.2 ± 0.5	2.6 ± 0.2^#^	4.0 ± 0.3^+^	2.9 ± 0.1^#^
%Fibrosis, %	N.A.	N.A.	59.4 ± 1.7	41.1 ± 1.4^#^	53.8 ± 2.7^+^	39.6 ± 1.4^#^
Vessel no., /HPF	N.A.	N.A.	3.5 ± 0.3	9.0 ± 0.7^#^	7.7 ± 0.3^#^	3.8 ± 0.3^+^
CD45, /HPF	N.A.	N.A.	7.3 ± 0.2	11.9 ± 1.2^#^	11.6 ± 2.1^#^	12.2 ± 1.7^#^
**Non-infarcted area**						
Myocyte size, mm	12.9 ± 0.1	14.7 ± 0.3*	15.4 ± 0.3*	17.2 ± 0.4*^#^	13.9 ± 0.1 +	16.6 ± 0.3*^#^
%Fibrosis, %	0.3 ± 0.1	0.3 ± 0.1	8.0 ± 1.0*	3.3 ± 0.7*^#^	8.6 ± 1.5*+	3.7 ± 0.6*^#^
Vessel no., /HPF	605 ± 40	613 ± 39	668 ± 43	735 ± 54	720 ± 21	665 ± 31
Vessel/myocyte ratio	2.2 ± 0.06	2.2 ± 0.06	2.2 ± 0.8	2.2 ± 0.6	2.3 ± 0.6	2.2 ± 0.4

MI, myocardial infarction; HPF high power field (x400); N.A., not applicable; *P < 0.05 *versus* saline-treated sham; #*P* < 0.05 *versus* saline-treated hearts with MI; ^+^*P* < 0.05 vers us G-CSF-treated hearts with MI

**Fig. 2:**
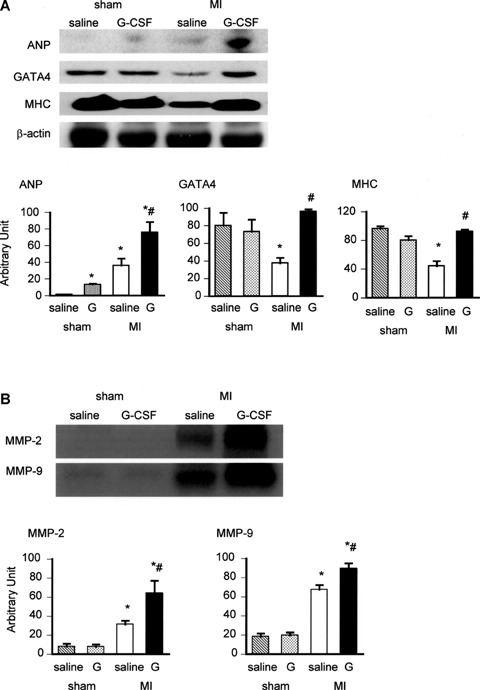
Western analysis of the effect of G-CSF on protein expression in hearts 4 weeks after MI. (**A**) Expression of ANP, GATA-4 and MHC. (**B**) Expression of MMP-2 and -9. *n*= 4 each for the saline-treated sham, the G-CSF-treated sham and the saline-treated MI groups; *n*= 5 for the G-CSF-treated MI group. *P < 0.05 *versus* sham-operated hearts treated with saline; ^#^*P* < 0.05 *versus* infarct-ed hearts treated with saline.

Cardiomyocyte hypertrophy was observed in the non-infarcted areas of hearts from control mice, most likely reflecting a compensatory mechanism. Somewhat unexpectedly, cardiomyocyte hypertrophy was even more pronounced in G-CSF-treated mice (Fig. 1D and Table 2). G-CSF also caused cardiomyocyte hypertrophy in sham-operated mice with normal cardiac function, suggesting that this hypertrophy was not simply a compensatory response. Ultrastructurally, salvaged cardiomyocytes from both the control and G-CSF groups showed degenerative changes that included partial myofibrillar loss and mitochondriosis, but the extent of those changes was considerably smaller in G-CSF-treated mice (Fig. 1E). Expression of ANP, a marker of cardiac hypertrophy [13], was found augmented not only by induction of MI but also by G-CSF (Fig. 2A). In addition, cardiac expression of GATA-4, a key transcriptional factor regulating the expression of sarcomeric proteins such as MHC [14], was significantly diminished in infarcted hearts, but was significantly restored by the G-CSF treatment (Fig. 2A). Expression of MHC was also reduced in infarcted hearts and it, too, was restored by G-CSF.

Fibrosis was significantly reduced in both the infarcted and non-infarcted left ventricular walls of G-CSF-treated hearts (Fig. 1B and C and Table 2). Western analysis showed that both MMP-2 and -9 were significantly overexpressed in G-CSF-treated hearts, as compared to control hearts (Fig. 2B), which is consistent with an earlier report showing induction of MMP-2 and -9 by G-CSF [15].

### Cardiomyocyte apoptosis

**Fig. 3:**
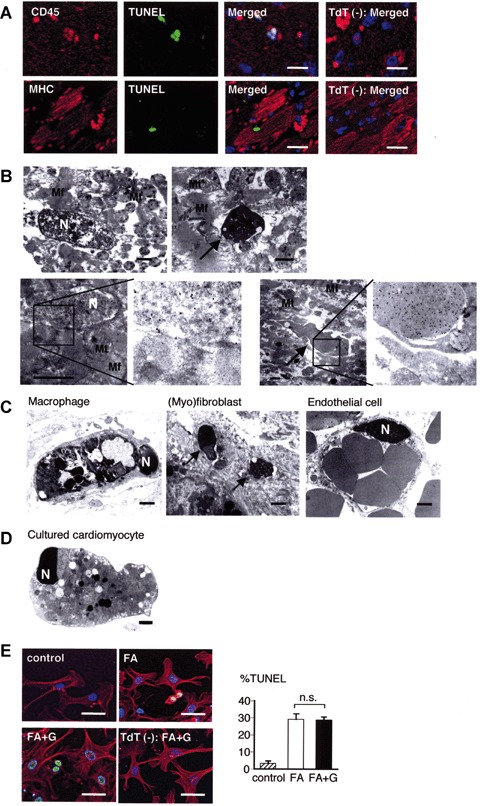
Analysis of cardiomyocyte and non-myocyte death. (**A**) TUNEL staining of CD45-positive leucocytes and MHC-positive cardiomyocytes on day 1 after MI. Bars, 10 μm. (**B**) Ultrastructure of infarcted myocardial tissue on day 1 after MI. Upper panels show a necrotic cardiomyocyte and an apoptotic inflammatory cell (arrow) that infiltrated into the necrotic cardiomyocyte. Lower left panels show an EM-TUNEL-positive necrotic cardiomyocyte with immunogold particle accumulation on the nucleus. Lower right panels show an ultrastructurally demonstrated apoptotic inflammatory cell (arrow) invading the necrotic cardiomyocyte; such true apoptotic cells present very strong EM-TUNEL positivity. N, nucleus; Mf, myofibril; Mt, mitochondria. Bars, 1 μm. (**C**) Ultrastructure of a macrophage, (myo)fibroblast and endothelial cell in the infarcted area showing typical apoptotic features 1 week after MI. Arrows indicate apoptotic bodies of which original cell type is assumed to be (myo)fibroblast because they are surrounded by dense fibrous tissue. Bars, 1 μm. (**D**) Ultrastructure of cultured neonatal cardiomyocytes subjected to Fas-mediated apoptosis. Bar, 1 μm. (**E**) Treatment with G-CSF did not affect the incidence of TUNEL-positivity among the cultured cardiomyocytes. Bars, 10 μm.

Because an anti-apoptotic effect on cardiomyocytes is reportedly one important cardioprotective mechanism of G-CSF [3], we assessed the incidence of cardiomyocyte apoptosis in the hearts 1 day, 1 week and 4 weeks after MI. Although TUNEL assay revealed the cells with fragmented DNA on day 1 after MI (57 ± 4 cells/HPF), almost all were non-myocytes (*e.g.* CD45-positive leucocytes); TUNEL-positive cardiomyocytes were extremely rare (Fig. 3A). Electron microscopy showed the apoptotic ultrastructure of affected leucocytes, but apoptotic cardiomyocytes were never seen; only necrotic features were observed in cardiomyocytes (Fig. 3B). Some leucocytes that had infiltrated into cardiomyocytes underwent apoptosis (Fig. 3B), and in EM-TUNEL assays some of the necrotic cardiomyocytes were positively labelled with immuno-gold particles, indicating fragmented DNA (Fig. 3B). These cells likely appeared to be TUNEL-positive and thus could be misinterpreted apoptotic at the immunofluorescence microscopy level. In any event, these cardiomyocytes were already necrotic 1 day after MI, so that G-CSF could not affect their rescue.

It also has been suggested that cardiomyocyte apoptosis within the salvaged area may contribute to the progression of post-MI heart failure [16]. For that reason, we next assessed the incidence of apoptosis in hearts at the subacute stage of infarction (1 week after MI). We found no TUNEL-positive cardiomyocytes at this stage. Moreover, electron microscopy revealed no cardiomyocytes with apoptotic ultra-structure, though apoptotic granulation tissue cells, including macrophages, myofibroblasts and endothelial cells were detected (Fig. 3C). Likewise, no TUNEL-positive cardiomyocytes were detected in hearts at the chronic stage of infarction (4 weeks after MI).

When we then directly investigated the anti-apoptotic effect of G-CSF using cultured neonatal cardiomyocytes in which apoptosis was induced by Fas stimulation (Fig. 3D) as previously reported [10], we found that G-CSF did not in any way modify the Fas-induced apoptosis (Fig. 3E).

### G-CSF receptor expression and downstream signal transduction

**Fig. 4:**
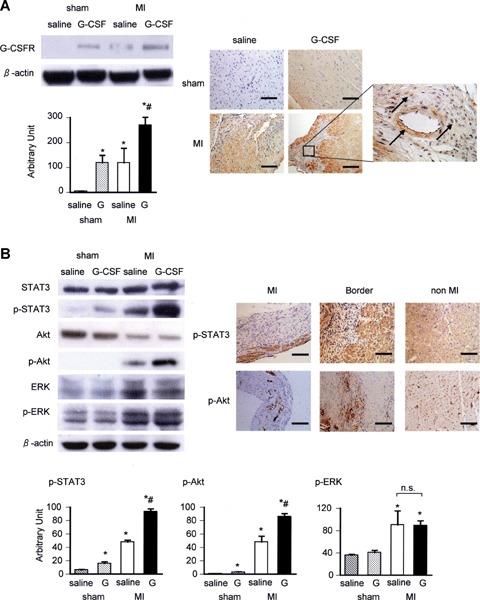
Expression of G-CSFR and its downstream signalling in hearts and its modulation by G-CSF. (**A**) Western and immunohistochemical analyses of G-CSFR expression. Immunohistochemistry shows localization of G-CSFRs on cardiomyocytes, interstitial cells and endothelial cells. Arrows in the right panel indicate G-CSFR-positive endothelial cells and interstitial cells. (**B**) Western analysis of the expression of STAT3, Akt, ERK and their phosphorylated forms (p-STAT3, p-Akt and p-ERK). Immunohistochemistry shows the distribution of p-STAT3 and p-Akt in the myocardium. Bars, 100 μm. *n*= 4 each for the saline-treated sham, the G-CSF-treated sham and the saline-treated MI groups; *n*= 5 for the G-CSF-treated MI group. *P < 0.05 *versus* sham-operated hearts treated with saline; #*P* < 0.05 *versus* infarcted hearts treated with saline.

Expression of G-CSF receptors (G-CSFRs) was augmented in post-MI hearts (Fig. 4A), which confirms the findings of an earlier study [3]. Moreover, the expression was significantly up-regulated in G-CSF-treated hearts, including the sham-operated hearts, which suggests G-CSF amplifies its effects by facilitating expression of its own receptor. Immunohistochemical analysis revealed that G-CSFRs were expressed on cardiomyocytes, interstitial cells and endothelial cells in the heart (Fig. 4A).

The ERK/mitogen-activated protein kinase (MAPK), JAK/STAT and PI3K/Akt pathways are known to serve as downstream mediators of G-CSFR signalling in haematopoietic cell lines and myocardium [3, 17, 18]. Western analysis of the expression levels of p-STAT3 and p-Akt showed that, following induction of MI, activation of STAT3 and Akt was more pronounced in the G-CSF-treated hearts than in controls, and that this enhancement even occurred in G-CSF-treated sham-operated hearts (Fig. 4B). On the other hand, G-CSF treatment did not affect expression of p-ERK, which was augmented in the infarcted hearts. p-STAT3 was detected immunohistochemically in cardiomyocytes and interstitial cells, while p-Akt was detected mainly in endothelial cells (Fig. 4B).

### Inhibition of JAK/STAT and PI3K/Akt signal activation

**Fig. 5:**
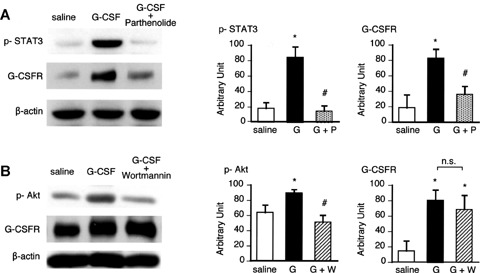
Comparison of the relative contributions made by STAT3 and Akt signalling to the up-regulation of G-CSFR in infarcted hearts 4 weeks after MI. Western analysis of the inhibition of STAT3 (**A**) and Akt (**B**) activation by parthenolide and wort-mannin, respectively, and their effects on myocardial G-CSFR expression. *n*= 4 each for the saline-treated group; *n*= 5 for the G-CSF-treated group; *n*= 3 each for the G-CSF and partheno-lide-treated group and G-CSF and wortmannin-treated group.

Given that activation of both the JAK/STAT and PI3K/Akt pathways was augmented by G-CSF treatment, we next evaluated their relative contributions to the beneficial effects in post-MI hearts. Parthenolide (1 mg/kg/day) or wortmannin (16 μg/kg/day) was given to the infarct-bearing mice at the same time G-CSF was administered, whereupon they were confirmed to respectively block the G-CSF-induced activation of STAT3 and Akt in the infarcted hearts (Fig. 5). Subsequent physiological evaluation of the survivors showed that parthenolide, but not wortmannin, abolished the beneficial effects of G-CSF on cardiac remodelling and function at the chronic stage (Table 1). Histologically, parthenolide, but not wortmannin, inhibited the reduction in MI area, the exaggeration of the cardiomyocyte hypertrophy/anti-degeneration, and the reduction in myocardial fibrosis brought about by G-CSF (Table 2). On the other hand, wortmannin, but not parthenolide, inhibited G-CSF-induced vascular proliferation in the infarcted area (Table 2).

**Fig. 6:**
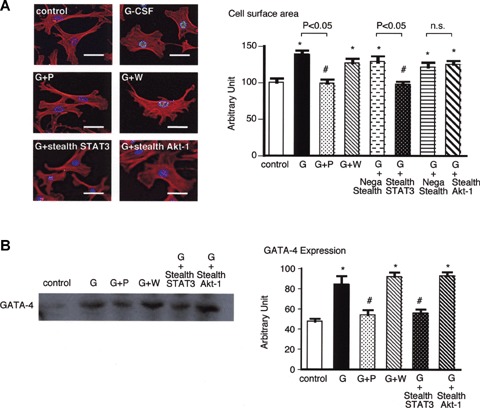
Comparison of the relative contributions made by STAT3 and Akt signalling to hypertrophy and GATA-4 expression in cultured cardiomyocytes. Effect of inhibiting STAT3 and Akt activation on rhG-CSF-induced hypertrophy (**A**) and GATA-4 expression (**B**) in cultured mouse neonatal cardiomyocytes. For examination of GATA-4 expression, both pharmacological inhibitors and siRNAs were used. Bars, 10 μm. Western analysis was done in triplicates for each group. *P < 0.05 *versus* sham-operated hearts treated with saline; ^#^*P* < 0.05 *versus* infarcted hearts treated with saline.

To more precisely evaluate the role of the JAK/STAT pathway in the hypertrophic/anti-degenerative effects of G-CSF, we performed an *in vitro* experiment using cultured cardiomyocytes. We found that both the surface area of cardiomyocytes and their expression of GATA-4 were significantly increased by the treatment with G-CSF, and those increases were completely abolished when the cells were pretreated with parthenolide or transfected with siRNA targeting STAT3, but not by wortmannin or siRNA targeting Akt-1 (Fig. 6).

## Discussion

The present study showed that G-CSF treatment at a small dose but for a long duration begun on the 2^nd^ day MI induced anti-degenerative effect among salvaged cardiomyocytes, reduced myocardial fibrosis and increased vessels in the heart. These phenotype changes were accompanied by up-regulation of GATA-4, MHC and MMP-2 and -9. Although treatment with G-CSF induced up-regulation of G-CSFR and activation of both STAT3 and Akt, our inhibition tests revealed that STAT3 inhibition, but not Akt inhibition, abolished beneficial effects of G-CSF and that anti-cardiomyocyte degenerative and anti-fibrotic effects were STAT3-dependent while angiogenetic effect was Akt-dependent. Cardiomyocyte apoptosis, on the other hand, seemed to play little role in the present model.

### Critical role of anti-cardiomyocyte degeneration and antifibrosis, both STAT-mediated, for the beneficial effects of G-CSF on the post-infarction heart

G-CSF induced cardiomyocyte hypertrophy with attenuated degeneration. This morphological observation is supported by observed increases in the expression of ANP, GATA-4 and MHC. The G-CSF-induced hypertrophy cannot be simply explained as a compensatory response because the hypertrophic reaction was more pronounced in G-CSF-treated hearts, which showed less severe heart failure than was seen in the untreated hearts. Furthermore, our *in vitro* study confirmed the direct hypertrophic/anti-degenerative effect of G-CSF on cultured cardiomyocytes. Hypertrophic growth of the myocardium is thought to preserve pump function, though prolongation of the hypertrophic state is a leading predictor of arrhythmias, sudden death and heart failure [19, 20]. Not all forms of cardiac hypertrophy are necessarily pathological, however, as athletic conditioning can stimulate heart growth without deleterious consequence [21]. In fact, transgenic mice that cardiospecifi-cally overexpressed STAT3 exhibited both hypertrophy and enhanced left ventricular function [22]. Notably in that regard, the cardiomyocyte hypertrophy observed in the present study was apparently mediated *via* the JAK/STAT pathway.

G-CSF significantly reduced cardiac fibrosis in the post-MI hearts, which is consistent with the earlier finding that G-CSF induces expression of MMP-2 and MMP-9 [15]. We confirmed the up-regulation of MMP expression in G-CSF-treated hearts, but the roles of MMPs in the failing heart are complicated: members of the MMP family of extracellular matrix proteases are generally up-regulated in failing hearts, and often their inhibition beneficially affect cardiac remodelling and function [23]. It is well known, however, that fibrosis, particularly interstitial fibrosis, often becomes excessive in failing hearts, accelerating cardiac remodelling and dysfunction, as was the case in the present model. Under those circumstances, an increase in MMP family proteins might exert a protective effect by catalyzing the degradation of the excessive collagen. Consistent with that idea, several studies have shown that inhibition of MMP causes cardiac failure [24]; that targeted deletion of MMP-9 enhances left ventricular remodelling and collagen accumulation caused by overexpression of MMP-2 and MMP-13 [25]; and that an increase in MMP-1 induced by hepatocyte growth factor has a beneficial effect on post-MI heart failure *via* its antifibrotic action [26]. Further studies are necessary to clarify the complex relations between MMP activation, myocardial fibrosis and heart failure.

On the othe hand, although our use of wortmannin to inhibit PI3K/Akt activation demonstrated that the PI3K/Akt pathway is critical for G-CSF-induced vascular proliferation in post-MI hearts, it revealed that vascular proliferation does not contribute to the improvement in post-MI cardiac remodelling and function.

We recently showed that G-CSF improves cardiac function in a murine model of doxorubicin (DOX)-induced non-ischemic cardiomyopathy [27]. In that model, G-CSF-induced molecular signalling downstream of G-CSFR differed substantially from that seen in the present MI model. ERK activation was suppressed in DOX-induced cardiomyopathy and was restored to a significant degree by G-CSF, which had no effect on STAT or Akt activation. Thus, G-CSF's downstream signalling *via* G-CSFR apparently varies in different models of heart failure.

### Insignificant role of Akt-mediated angiogenesis and anti-apoptosis for the beneficial effects of GCSF on the post-infarction heart

Harada *et al.* reported that G-CSF exerted an anti-apoptotic effect on cardiomyocytes that was accompanied by up-regulation of the anti-apoptotic proteins Bcl-2 and Bcl-xL [3]. In the present model, we detected no apoptotic cardiomyocytes 1 day after MI. Ultrastructurally, all cardiomyocytes in the infarcted area exhibited necrotic features, some of which appeared as false-positives in EM-TUNEL assays, which is consistent with our earlier findings [12]. This means that the infarcted myocardium was not the target of the therapeutic effect of G-CSF at the time of its administration. In addition, G-CSF did not affect the induction of apoptosis by Fas stimulation in cultured cardiomyocytes. We also failed to detect apoptotic cardiomyocytes in the salvaged myocardial tissue at the subacute and chronic stages of infarction. Overall, these findings indicate that attenuation of cardiomyocyte apoptosis is unlikely to be a major mechanism underlying the beneficial effects of G-CSF in the present model. Nevertheless, G-CSF reduced the size of infarct scar. Its precise mechanism is unknown. However, G-CSF significantly reduced cardiac fibrosis in the G-CSF significantly reduced cardiac fibrosis in the post-MI hearts and an infarct scar consists mainly of fibrous tissue. Thus, reduction of infarct size by G-CSF might, at least in part, be explained by this fibrosis reducing effect.

### Study limitations

G-CSF was originally reported to enhance post-infarct myocardial regeneration by mobilized bone marrow-derived cells to the myocardium [1]. This effect is still controversial [1–6] and we did not examine it in the present study. This study did not address myocardial regeneration by resident cardiac stem cells, either [28] and a possible effect of G-CSF on it remains unresolved.

### Clinical implications

Two clinical implications may be drawn from the present findings. First, G-CSF was started 1 day after the onset of acute MI and found to be effective to the post-infarct process, at which an appropriate time window already passed for coronary reperfusion therapy to protect myocardium from ischemic death. This suggests a late treatment with G-CSF is still effective for patients with MI. Second, the daily dose of G-CSF used in the present study was substantially low (approximately 1/10 of the earlier studies). This may imply a possibility that doses of G-CSF could be set smaller reduced than usual when applying to the patients with acute MI.
